# Comparison of standardized uptake values between ^99m^Tc-HDP SPECT/CT and ^18^F-NaF PET/CT in bone metastases of breast and prostate cancer

**DOI:** 10.1186/s13550-019-0475-z

**Published:** 2019-01-24

**Authors:** Samuli Arvola, Ivan Jambor, Anna Kuisma, Jukka Kemppainen, Sami Kajander, Marko Seppänen, Tommi Noponen

**Affiliations:** 1 Department of Clinical Physiology and Nuclear Medicine, Turku University Hospital, Kiinamyllynkatu 4-8, 20521 Turku, Finland; 2 Department of Diagnostic Radiology, University of Turku, Turku, Finland; 3 Department of Oncology and Radiotherapy, University of Turku, Turku, Finland; 4 Turku PET Centre, Turku, Finland; 5 Department of Medical Physics, Turku University Hospital, Turku, Finland

**Keywords:** Quantitative SPECT, SUV SPECT, SUV comparison, SUV ratio, Bone metastases

## Abstract

**Background:**

Despite recent technological advances allowing for quantitative single-photon emission computed tomography (SPECT), quantitative SPECT has not been widely used in the clinical practice. The aim of this study is to evaluate the feasibility of quantitative SPECT for measuring metastatic bone uptake in breast and prostate cancer by comparing standard uptake values (SUVs) measured with ^99m^Tc-HDP SPECT/CT and ^18^F-NaF PET/CT.

**Methods:**

Twenty-six breast and 27 prostate cancer patients at high risk of bone metastases underwent both ^99m^Tc-HDP SPECT/CT and ^18^F-NaF PET/CT within 14 days of each other. The SPECT and PET data were reconstructed using ordered-subset expectation-maximization algorithms achieving quantitative images. Metastatic and benign skeletal lesions visible in both data sets were identified, and their maximum, peak, and mean SUVs (SUV_max_, SUV_peak_, and SUV_mean_) were determined. SUV ratios (SUVRs) between the lesions and adjacent normal appearing bone were also calculated. Linear regression was used to evaluate the correlations between the SUVs of SPECT and PET and Bland-Altman plots to evaluate the differences between the SUVs and SUVRs of SPECT and PET.

**Results:**

A total of 231 skeletal lesions, 129 metastatic and 102 benign, were analyzed. All three SUV measures correlated very strongly between SPECT and PET (*R*^*2*^ ≥ 0.80, *p* < 0.001) when all lesions were included, and the PET SUVs were significantly higher than SPECT SUVs (*p* < 0.001). The median differences were 21%, 12%, and 19% for SUV_max_, SUV_peak_, and SUV_mean_, respectively. On the other hand, the SUVRs were similar between SPECT and PET with median differences of 2%, − 9%, and 2% for SUVR_max_, SUVR_peak_, and SUVR_mean_, respectively.

**Conclusion:**

The strong correlation between SUVs and similar SUVRs of ^99m^Tc-HDP SPECT/CT and ^18^F-NaF PET/CT demonstrate that SPECT is an applicable tool for clinical quantification of bone metabolism in osseous metastases in breast and prostate cancer patients.

## Background

Single-photon emission computed tomography (SPECT) has conventionally been used in a non-quantitative manner, i.e., the images have been interpreted using relative intensity values instead of absolute values of tracer concentration [1, 2]. However, the wide acceptance of integrated SPECT/CT scanners and the development of iterative reconstruction algorithms have made the clinical use of quantitative SPECT possible [3, 4]. Several applications for quantitative SPECT have been suggested but they have not been widely adopted in clinical routine use yet [1].

SPECT/CT with ^99m^Tc-labeled diphosphonates, such as ^99m^Tc-hydroxyethylene diphosphonate (HDP), is increasingly used for the detection of bone metastases in breast and prostate cancer patients [5]. The sensitivity and specificity of SPECT/CT are superior compared to conventional bone scintigraphy or SPECT alone [6, 7]. ^18^F-NaF PET/CT is an even more sensitive method than ^99m^Tc-diphosphonate SPECT/CT [6, 8], and PET is inherently quantitative [9]. However, ^18^F-NaF PET/CT is rather expensive and has limited availability [2, 6].

Quantitative SPECT and PET allow the calculation of standardized uptake values (SUVs), which can be utilized for longitudinal disease assessment and interpatient comparison [10]. Feasibility of quantitative bone SPECT has been previously demonstrated by reporting skeletal SUVs [2, 11–14]; however, a direct comparison to a standard reference method, such as PET/CT, has not yet been performed. ^18^F-NaF PET/CT has been validated using bone biopsy findings [15, 16] and proven to be repeatable in patients with bone metastases [17, 18], therefore being a potential standard method for the diagnosis of metastatic bone disease.

In this study, our aim is to explore the correlation between SUVs measured by quantitative ^99m^Tc-HDP SPECT/CT and those measured by ^18^F-NaF PET/CT in the evaluation of metastatic bone uptake in breast and prostate cancer patients.

## Methods

### Study design and patients

This study included the same patients as a previously published study by Jambor et al. (NCT01339780, ClinicalTrials.gov) [6]. Twenty-six breast and 27 prostate cancer patients at high risk for bone metastases were prospectively enrolled between February 2011 and March 2013. Each patient gave written informed consent, and the study was approved by the local ethics committee. Patients were included if they had localized pain in the skeletal area suggesting bone metastases, suspicious laboratory findings (elevated alkaline phosphates, elevated PSA, or high PSA doubling time after prostatectomy), or suspicious histopathologic findings (stage N3a or higher in breast cancer patients, stage T3a or higher, and/or Gleason score of 4 + 3 or higher in prostate cancer patients). All patients underwent ^99m^Tc-HDP SPECT/CT, ^18^F-NaF PET/CT, and 1.5-T whole-body magnetic resonance imaging (MRI), including diffusion-weighted imaging (DWI), within 14 days in a varying order. The median (range) interval between ^99m^Tc-HDP SPECT/CT and ^18^F-NaF PET/CT was 3 (1–14) days [6].

### ^99m^Tc-HDP SPECT/CT

The patients received intravenous injection of 672 ± 21 (mean ± SD) MBq of ^99m^Tc-HDP. The SPECT scans were performed 180 ± 24 (mean ± SD) min after the injection using a Symbia T6, True Point SPECT/CT scanner (Siemens Healthcare, Erlangen, Germany) with the following parameters: low-energy high-resolution collimators, three bed positions, 180 projections over 360° with 9-s acquisition time per view, 128 × 128 matrix, 4.8 × 4.8 mm pixel size, and 4.8-mm slice thickness. Low-dose CT scans were acquired with 130 kV and 10 effective mAs. The CT data were reconstructed using a smooth attenuation-correction kernel B08s and a sharp bone kernel B65s.

The SPECT data were reconstructed with HybridRecon-Oncology SUV SPECT (version 1.3, HERMES Medical Solutions AB, Stockholm, Sweden) using the three-dimensional (3D) ordered-subset expectation-maximization (OSEM) algorithm with 10 iterations and 15 subsets [19]. A relatively high number of iterations was used to increase the spatial resolution of SPECT images closer to the spatial resolution of PET. The reconstruction included corrections for decay, attenuation, scatter, and collimator response. Attenuation correction was based on attenuation coefficient maps derived from the B08s CT images [20]. Scatter correction was performed with a Monte Carlo simulation using 10^5^ simulated photons and two scatter update iterations [21]. The collimator response was corrected using a Gaussian diffusion model [22]. The images were postfiltered with a Gaussian filter of 7-mm full width at half maximum (FWHM).

### SPECT scanner calibration

The SPECT scanner was calibrated by scanning a uniform Jaszczak phantom (Data Spectrum Corporation, Durham, NC, USA) without any inserts inside and filled with water and 150 MBq of ^99m^Tc-pertechnetate. A conversion factor to convert the reconstructed counts into units of activity concentration (Bq/ml) was calculated as the ratio between true activity and reconstructed counts in a homogeneous volume of interest (VOI). The resulting conversion factor was 0.107 kBq/cps.

### ^18^F-NaF PET/CT

The patients received intravenous injection of 209 ± 7 (mean ± SD) MBq of ^18^F-NaF. Whole-body PET scans were performed 64 ± 6 (mean ± SD) min after the injection using a Discovery VCT PET/CT or Discovery PET/CT 690 scanner (GE Healthcare, Milwaukee, WI, USA). A static emission scan from feet to skull vertex was performed with 3- (VCT scanner) or 2-min (690 scanner) acquisition time per bed position.

The PET data were corrected for decay, attenuation, scatter, random coincidences, and dead time. The data acquired with Discovery VCT PET/CT were reconstructed in a 128 × 128 matrix with a pixel size of 5.47 × 5.47 mm and slice thickness of 3.27 mm using VUE Point HD (GE Healthcare, Milwaukee, WI, USA), a fully 3D OSEM algorithm with 2 iterations and 28 subsets. Discovery PET/CT 690 data were reconstructed in a 192 × 192 matrix with a pixel size of 3.65 × 3.65 mm and a slice thickness of 3.27 mm using VUE Point FX-Sharp IR (GE Healthcare, Milwaukee, WI, USA), also a fully 3D OSEM algorithm with 2 iterations and 24 subsets incorporating time-of-flight and point-spread function information. The images acquired with Discovery VCT PET/CT and Discovery PET/CT 690 were postfiltered with Gaussian filters of 7- and 9-mm FWHM, respectively, to lower the spatial resolution of PET closer to the spatial resolution of SPECT.

The PET scanners were quarterly calibrated following the EANM/EARL FDG-PET/CT accreditation procedure [23].

### Image analysis

Image analysis was performed using HybridViewer (version 2.6, HERMES Medical Solutions AB, Stockholm, Sweden), which converted values of activity concentration into SUVs according to the following equation:

1 $$ \mathrm{SUV}=\frac{\mathrm{voxel}\ \mathrm{activity}\ \mathrm{concentration}\ \left(\frac{\mathrm{Bq}}{\mathrm{ml}}\right)\times \mathrm{patient}\ \mathrm{weight}\ \left(\mathrm{g}\right)}{\mathrm{decay}\ \mathrm{corrected}\ \mathrm{injected}\ \mathrm{activity}\ \left(\mathrm{Bq}\right)}\times 1\ \frac{\mathrm{ml}}{\mathrm{g}}. $$

**Fig. 1:**
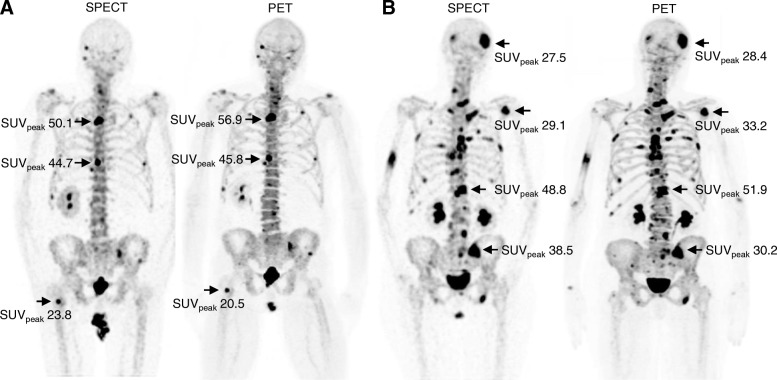
Maximum intensity projections of ^99m^Tc-HDP SPECT and ^18^F-NaF PET scans acquired (**a**) from the same 66-year-old prostate cancer patient and (**b**) from the same 75-year-old breast cancer patient. SUVs_peak_ are shown for most active lesions. The SPECT scan of the prostate cancer patient was acquired 2 days before the corresponding PET scan and the SPECT scan of the breast cancer patient 5 days after the corresponding PET scan

Skeletal lesions visible in both SPECT and PET images were classified into benign and metastatic by the consensus of two experienced nuclear medicine physicians (Fig. 1). Localization CT images of PET/CT and SPECT/CT and anatomical and DWI MRI images were used to verify the lesion classification according to corresponding morphologic findings [6].

For analyzing purposes, the identified lesions were first segmented from PET images using a threshold of SUV > 15, which was lowered if the resulting VOI was clearly smaller than the area of increased uptake. Next, the same lesions were segmented from SPECT images using SUV thresholds that resulted in VOIs with the volumes similar to those in PET images.

Maximum, peak, and mean SUVs (SUV_max_, SUV_peak_, and SUV_mean_) were calculated from the VOIs. SUV_max_ was determined as the SUV of the most active voxel within the VOI, SUV_peak_ as the average SUV of a 1-cm^3^ cube positioned within the lesion such that the enclosed average SUV was maximized, and SUV_mean_ as the average SUV of voxels in the specific VOI. The cubic VOI of SUV_peak_ could extend outside the lesion VOI in small lesions, which sometimes caused SUV_peak_ being lower than SUV_mean_.

Background bone activity was determined separately for every lesion by placing five to ten circular regions of interest (ROIs) of 1-cm diameter on adjacent normal-appearing bone tissue. These ROIs were summed to form background VOI, whose mean SUV (SUV_mean,bg_) was calculated. Maximum, peak, and mean SUV ratios (SUVR_max_, SUVR_peak_, and SUVR_mean_) of the lesion were calculated by dividing the SUV_max_, SUV_peak_, and SUV_mean_ of the lesion by the corresponding SUV_mean,bg_.

### Statistical analyses

Statistical analyses were performed using MATLAB (version R2016A, The MathWorks, Natick, MA, USA) and SPSS (version 24, IBM Corp., Armonk, NY, USA) software. Scatter plots and linear regression models were used to evaluate the overall correlation between SUVs of SPECT and PET data, and the Bland-Altman plots were created to evaluate the agreement between SUVs and SUVRs of SPECT and PET data. In the Bland-Altman plots, the mean difference and 95% limits of agreement (LOA) were estimated using the median and the 2.5th and 97.5th percentiles of the differences because the differences were not normally distributed according to the Shapiro-Wilk test.

The Wilcoxon signed rank test was used to determine whether the SUVs and SUVRs of SPECT and PET were statistically different, and the Mann-Whitney *U* test was used to determine whether the SUVs and SUVRs of metastatic lesions were significantly higher than the SUVs and SUVRs of benign lesions. These nonparametric statistical methods were employed because the SUV and SUVR data were not normally distributed. Similarly, median and interquartile range (IQR or middle 50%) were used to report SUVs.

## Results

A total of 231 skeletal lesions, 129 metastatic and 102 benign, were analyzed from 46 patients. The average SUV thresholds used for segmentation were 10.7 for SPECT and 12.9 for PET. Segmented lesion volumes varied from 0.55 to 60 cm^3^ with a median of 2.0 cm^3^, and the volumes were practically the same for the corresponding SPECT and PET lesions due to the segmentation method used (the volume difference ranged from 0 to 13% with a median of 2%). The number of analyzed lesions per patient varied from 0 to 21 with a median of 3. Metastatic lesions were analyzed from 18 patients and benign lesions from 37 patients. Seven patients had no identified lesions. Patient and lesion characteristics are listed in Table 1.

**Table 1:** Patient and lesion characteristics

	Breast cancer	Prostate cancer
Patients, *n*	26	27
Median age (range), years	61 (46–76)	67 (52–79)
Median height (range), cm	167 (158–175)	178 (169–186)
Median weight (range), kg	70 (56–99)	87 (60–110)
Metastatic lesions, *n*		
0	16	19
1–5	4	6
6–10	3	1
11–20	3	1
Benign lesions, *n*		
0	5	2
1–5	14	22
6–10	3	2
11–20	4	1

**Fig. 2:**
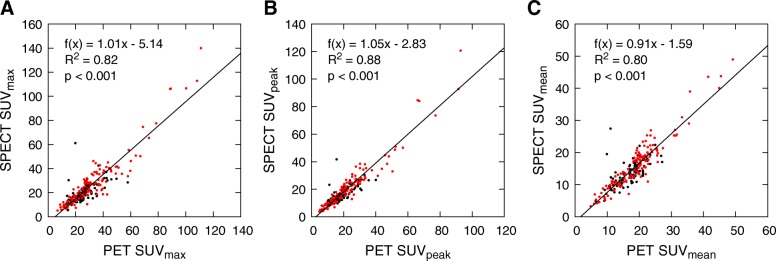
Scatter plots of **a** SUV_max_, **b** SUV_peak_, and **c** SUV_mean_ from SPECT and PET data for all lesions. Metastatic lesions are marked in red and benign in black. Regression lines with slopes, *y*-intercepts, and coefficients of determination (*R*^2^) have been calculated using the method of least squares

Scatter plots with regression lines for SUVs are shown in Fig. 2. Correlations between SPECT and PET SUVs are strong and statistically significant (*R*^*2*^ ≥ 0.80, *p* < 0.001), and PET SUVs are significantly higher than SPECT SUVs (*p* < 0.001). The regression lines have slopes close to one in all SUV analyses. *y*-intercepts suggest a constant bias between all SPECT and PET SUVs which is largest for SUV_max_ and smallest for SUV_mean_ (Fig. 2).

Metastatic lesions have generally higher SUVs than benign lesions, but the SUV distributions of benign and metastatic lesions overlap greatly (Fig. 2). According to the Mann-Whitney *U* test, the metastatic lesions had significantly higher PET SUV_max_ (*p* < 0.05), PET SUV_peak_ (*p* < 0.01), SPECT SUV_peak_ (*p* < 0.01), and SPECT SUV_mean_ (*p* < 0.01) than the benign lesions, but PET SUV_mean_ and SPECT SUV_max_ were not significantly different between benign and metastatic lesions. Therefore, SUV alone could not be used to differentiate metastatic and benign lesions.

**Fig. 3:**
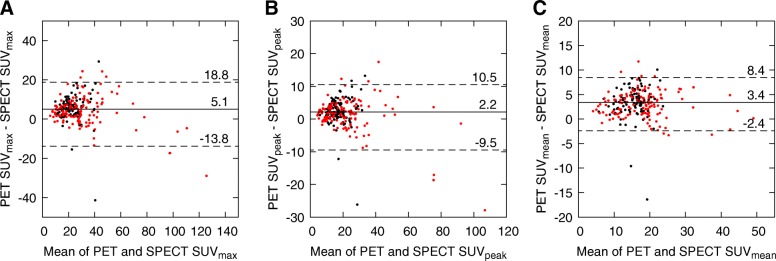
Bland-Altman plots of **a** SUV_max_, **b** SUV_peak_, and **c** SUV_mean_ from SPECT and PET data for all lesions. The *x*-axis represents the mean of SPECT and PET SUVs and the *y*-axis the difference between SPECT and PET SUVs. Metastatic lesions are marked in red and benign in black. Solid lines with numerical values denote median differences and dotted lines with values denote 95% LOA

The Bland-Altman plots of SUV_max_, SUV_peak_, and SUV_mean_ from SPECT and PET data are shown in Fig. 3. Among these analyses, SUV_peak_ reveals the smallest systematic difference and all SUV measures show only a moderate range of deviation and LOA for differences. The median differences (and LOA) normalized with respect to SPECT SUVs are 21% (− 26–53%), 12% (− 28–39%), and 19% (− 11–45%) for SUV_max_, SUV_peak_, and SUV_mean_, respectively. The effect of lesion volume on the SUV differences between SPECT and PET is shown in Table 2. The differences are smaller in larger lesions.

**Table 2:** Median (IQR) differences between SUVs of SPECT and PET for lesions in different size categories. The differences are normalized with respect to SPECT SUVs

Size category	n	SUV_max_	SUV_peak_	SUV_mean_
0.55–1.21 cm^3^	58	41% (− 22–162%)	17% (− 20–87%)	30% (− 12–111%)
1.32–1.98 cm^3^	58	24% (− 24–89%)	11% (− 39–52%)	19% (− 34–63%)
1.98–3.97 cm^3^	58	17% (− 45–106%)	7% (− 45–76%)	17% (− 39–90%)
3.97–60.31 cm^3^	57	11% (− 29–83%)	6% (− 26–52%)	17% (−14–51%)

**Fig. 4:**
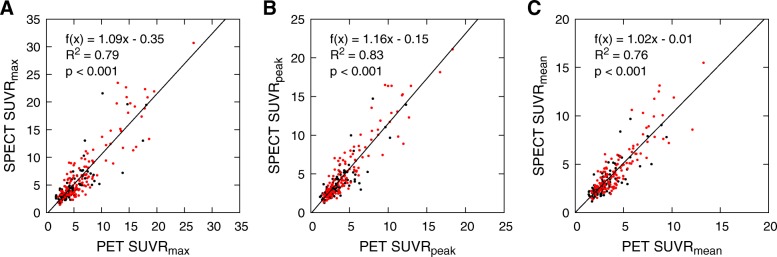
Scatter plots of **a** SUVR_max_, **b** SUVR_peak_, and **c** SUVR_mean_ from SPECT and PET data for all lesions. Metastatic lesions are marked in red and benign in black. Regression lines with slopes, *y*-intercepts, and coefficients of determination (*R*^2^) have been calculated using the method of least squares

SUVs_mean,bg_ for different skeletal sites are listed in Table 3, and scatter plots with regression lines for SUVRs are shown in Fig. 4. Correlations between SPECT and PET SUVRs are slightly weaker than the correlations between corresponding SUVs, but the regression lines are closer to the line of equality as their *y*-intercepts are almost 0. Even though the SUVR distributions of benign and metastatic lesions overlap like the SUV distributions, all SUVR measures are significantly different (*p* < 0.001) between benign and metastatic lesions.

**Table 3:** SUVs_mean,bg_ from normal appearing bone at different skeletal sites

Skeletal site	PET SUV median (IQR)	SPECT SUV median (IQR)	PET SUV–SPECT SUV median (IQR)^*^
Skull	2.6 (2.1–5.0)	2.0 (1.8–2.6)	0.3 (0.1–0.8) (*p* < 0.01)
Spine	7.4 (6.6–8.7)	6.1 (4.7–6.8)	1.7 (0.6–2.8) (*p* < 0.001)
Rib cage	3.0 (2.4–4.0)	2.5 (2.0–3.1)	0.7 (−0.1–1.0) (*p* < 0.001)
Pelvis	5.5 (4.7–6.6)	4.7 (4.1–6.0)	0.6 (−0.2–1.3) (*p* < 0.01)
Limbs	3.8 (2.9–4.9)	3.2 (2.1–4.3)	0.7 (0.1–1.0) (*p* < 0.01)

*IQR* interquartile range

^*^p value denotes at which level a median difference is significantly different from 0 according to the Wilcoxon signed rank test

**Fig. 5:**
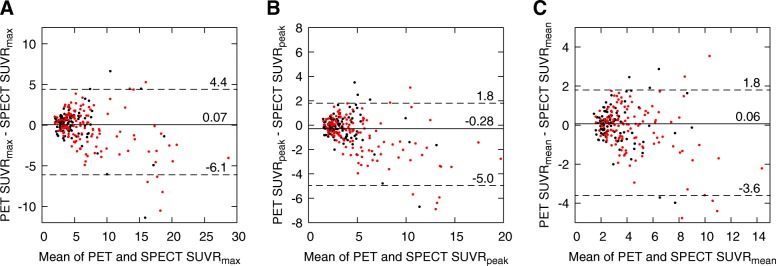
Bland-Altman plots of **a** SUVR_max_, **b** SUVR_peak_, and **c** SUVR_mean_ from SPECT and PET data for all lesions. The *x*-axis represents the mean of SPECT and PET SUVRs and the *y*-axis the difference between SPECT and PET SUVRs. Metastatic lesions are marked in red and benign in black. Solid lines with numerical values denote median differences and dotted lines with values denote 95% LOA

The Bland-Altman plots for SUVR measures are shown in Fig. 5. SUVRs are even more similar than SUVs in Fig. 3 between SPECT and PET data because PET images have higher background SUVs than SPECT images (Table 3). According to the Wilcoxon signed rank test, only SUVRs_peak_ were statistically different between SPECT and PET data. The median differences (and LOA) normalized with respect to SPECT SUVRs are 2% (− 45–88%), − 9% (− 47–59%), and 2% (− 42–65%) for SUVR_max_, SUVR_peak_, and SUVR_mean_, respectively.

## Discussion

We evaluated the correlation and similarity of SUVs and SUVRs between ^99m^Tc-HDP SPECT and ^18^F-NaF PET in different benign skeletal lesions and bone metastases of breast and prostate cancer patients. To our knowledge, this is the first study to report SUVs and SUVRs of ^99m^Tc-diphosphonate SPECT using ^18^F-NaF PET as a reference standard. Strong correlations were shown between SUVs and similarities between SUVRs of these two methods. Previous clinical research has mainly reported ^99m^Tc-diphosphonate SPECT SUVs in different skeletal structures without direct reference values [2, 11–14]. Our results demonstrate that ^99m^Tc-HDP SPECT SUVs and SUVRs reflect the metabolic activity of lesions similarly to ^18^F-NaF PET SUVs and SUVRs; thus, SPECT measures can potentially be used to clinically evaluate bone metastases.

In all our analyses, PET SUVs were systematically higher than SPECT SUVs. Due to poorer spatial resolution, underestimation of SUV caused by the partial volume effect is more significant in SPECT than PET [1]. However, a higher number of OSEM iterations in SPECT and wide Gaussian filters in PET were used to compensate for this resolution difference between SPECT and PET. This compensation roughly halved the absolute differences between the SUVs of SPECT and PET and reduced the differences in SUVR_max_ and SUVR_mean_ between SPECT and PET by 17 and 6 percentage units, respectively. The difference in SUVR_peak_ increased by 6 percentage units.

After the compensation, the SUVs of PET were still significantly higher, even in background bone where no significant partial volume effect is expected. This suggests that a higher fraction of ^18^F-NaF than ^99m^Tc-HDP is extracted from blood by bone. This difference is most likely caused by the different pharmacokinetics of ^99m^Tc-HDP and ^18^F-NaF. For instance, blood protein binding hinders the extraction of ^99m^Tc-diphosphonates but does not affect the ^18^F-NaF extraction [24–26]. The SUV differences were also larger in smaller lesions, which suggests that some difference was caused by the different spatial resolutions of SPECT and PET systems even after the compensation.

In this study, SUVRs were not significantly different between SPECT and PET. Previously, the SUVR has been used mainly in the interpretation of brain PET. The SUV differences of lesions between different skeletal areas are partly caused by the differences in SUVs_mean,bg_ between these areas, the highest SUVs and SUVs_mean,bg_ being in the spine and the lowest in the skull. SUVs of lesions and their corresponding SUVs_mean,bg_ cause that SUVRs are more similar than corresponding SUVs between different skeletal sites, and being also very similar between SPECT and PET. The similarity of SUVRs between SPECT and PET arises the question whether SUVRs should be used instead of SUVs for the evaluation of metastatic uptake, both in quantitative PET and especially in quantitative SPECT. SUVRs can be calculated without scanner calibration or information on the patient weight or injected dose, making them technically easier to accomplish than SUVs and equal to target-to-background ratios. However, reliable calculation of SUVs_mean,bg_ is manually laborious and sensitive to inter-observer variability; thus, the procedure should ideally be automatized.

The typical SUV measure in PET has been SUV_max_ due to its simplicity and nonexistent inter-observer variability [27]. However, SUV_max_ is subject to noise and statistical variations in data [28]. Consequently, SUV_peak_ and SUV_mean_ have gained interest as robust alternatives to SUV_max_. Also in this study, SUV_peak_ and SUV_mean_ were more similar than SUV_max_ between SPECT and PET. The strongest correlation and smallest systematic difference between SPECT and PET were in SUV_peak_, which is measured as an average SUV of voxels in a 1-cm^3^ cube. Due to finite voxel size, the volumes of cubes were not exactly 1 cm^3^. In fact, the SUV_peak_ of SPECT was calculated from a 26% smaller volume than the SUV_peak_ of PET. This somewhat compensates for the generally higher SUVs of PET, which may partly result in the smallest difference in SUV_peak_. The SPECT SUV_peak_, and SUV_max_ as well, may have also been slightly overestimated due to collimator response correction artifact [29]. These same factors increasing SPECT SUVs_peak_ may also cause SPECT SUVRs_peak_ to be higher than PET SUVRs_peak_ resulting in the largest systematic difference among our SUVR measures. However, the absolute differences we found between the SUVRs of SPECT and PET were generally very small.

The strong correlation of SUVs and very similar SUVRs between ^99m^Tc-HDP SPECT and ^18^F-NaF PET suggest that the SUVs and SUVRs of ^99m^Tc-HDP SPECT are valid and feasible for research and clinical use. These results encourage SPECT SUVs to be utilized in clinical trials and follow-up studies to decrease inter-observer variability and to standardize SPECT results between patients, imaging systems, and clinical centers. Even though ^18^F-NaF PET/CT is a more sensitive method than ^99m^Tc-HDP SPECT/CT, the cost of ^18^F-NaF PET/CT is three- to fourfold that of ^99m^Tc-HDP SPECT/CT [6], making ^99m^Tc-HDP SPECT/CT the more cost-effective modality for the evaluation of metastatic bone uptake in breast and prostate cancer. SPECT has also wider availability than PET in most countries [2]. The SUV regression lines have slopes so close to one that the SUVs of SPECT and PET would even be equal in some cases if a small positive bias was added into SPECT SUV. However, because the Bland-Altman plots show rather wide 95% LOA, PET SUVs cannot be directly converted into SPECT SUVs. Therefore, usage of SPECT as follow-up for PET is not recommended.

The requirements for producing quantitative SPECT and PET data are the same. The most important ones are scanner calibration and a reconstruction algorithm correcting for photon attenuation and scatter within the object [1]. The calculation of reliable SUVs also requires accurate measurements of prepared activity, preparation time, injection time, syringe residual activity, time of residual activity measurement, and patient weight.

The main limitations of our study are the relatively small number of patients with bone metastases and the lack of accurate injected activities used in SPECT. We have afterwards measured the distribution of injected activities from a sample of 38 syringes used for bone scans at our department. Our average injected activity should be 670 MBq. In that estimation, the injected activity was calculated as the activity of full syringe subtracted by the residual activity, and it had an average of 672 MBq and SD of 21 MBq. This average injected activity was eventually used in the SPECT SUV calculations of this study. We assume that the inaccuracy in the assessment of injected activity results in a maximum random error of 7% (= 2 SDs) in the individual SPECT SUV calculations and no error in the SPECT SUVR calculations. This error in SPECT SUV calculations is relatively small compared to SUV differences between SPECT and PET, so it does not have a significant influence on the statistical differences between SPECT and PET SUVs.

## Conclusion

The SUVs of ^99m^Tc-HDP SPECT/CT and ^18^F-NaF PET/CT correlate strongly and SUVRs are very similar, showing that SPECT SUVs are feasible for uptake measurements in bone metastases of breast and prostate cancer. SUVs and SUVRs could be used to expand the visual analysis of skeletal SPECT especially in follow-up studies. The SUVs of ^99m^Tc-HDP SPECT/CT are slightly lower than the SUVs of ^18^F-NaF PET/CT.

## Abbreviations

3D: Three-dimensional
CT: Computed tomography
DWI: Diffusion-weighted imaging
FWHM: Full width at half maximum
HDP: Hydroxyethylene diphosphonate
IQR: Interquartile range
LOA: Limits of agreement
MRI: Magnetic resonance imaging
OSEM: Ordered-subset expectation-maximization
PET: Positron emission tomography
PSA: Prostate-specific antigen
ROI: Region of interest
SD: Standard deviation
SPECT: Single-photon emission computed tomography
SUV: Standardized uptake value
SUVR: Standard uptake value ratio
VOI: Volume of interest
